# NEMPD: a network embedding-based method for predicting miRNA-disease associations by preserving behavior and attribute information

**DOI:** 10.1186/s12859-020-03716-x

**Published:** 2020-09-10

**Authors:** Bo-Ya Ji, Zhu-Hong You, Zhan-Heng Chen, Leon Wong, Hai-Cheng Yi

**Affiliations:** 1 Xinjiang Technical Institutes of Physics and Chemistry, Chinese Academy of Sciences, Urumqi, 830011 China; 2 University of Chinese Academy of Sciences, Beijing, 100049 China

**Keywords:** miRNA-disease associations, Heterogeneous network, GraRep, Random Forest

## Abstract

**Background:**

As an important non-coding RNA, microRNA (miRNA) plays a significant role in a series of life processes and is closely associated with a variety of *Human* diseases. Hence, identification of potential miRNA-disease associations can make great contributions to the research and treatment of *Human* diseases. However, to our knowledge, many existing computational methods only utilize the single type of known association information between miRNAs and diseases to predict their potential associations, without focusing on their interactions or associations with other types of molecules.

**Results:**

In this paper, we propose a network embedding-based method for predicting miRNA-disease associations by preserving behavior and attribute information. Firstly, a heterogeneous network is constructed by integrating known associations among miRNA, protein and disease, and the network representation method Learning Graph Representations with Global Structural Information (GraRep) is implemented to learn the behavior information of miRNAs and diseases in the network. Then, the behavior information of miRNAs and diseases is combined with the attribute information of them to represent miRNA-disease association pairs. Finally, the prediction model is established based on the Random Forest algorithm. Under the five-fold cross validation, the proposed NEMPD model obtained average 85.41% prediction accuracy with 80.96% sensitivity at the AUC of 91.58%. Furthermore, the performance of NEMPD is also validated by the case studies. Among the top 50 predicted disease-related miRNAs, 48 (breast neoplasms), 47 (colon neoplasms), 47 (lung neoplasms) were confirmed by two other databases.

**Conclusions:**

The proposed NEMPD model has a good performance in predicting the potential associations between miRNAs and diseases, and has great potency in the field of miRNA-disease association prediction in the future.

## Background

MicroRNAs (miRNAs) are a kind of endogenous non-coding RNA with a length of ~ 22 nt, which regulates the expression of target mRNAs by controlling the expression of target genes through sequence complementary pairing [1]. The sequence of miRNA is very short, and it is only expressed in specific tissues or cells at specific stages, so miRNAs are not well known to people before and usually called dark matter in life [2]. In 1993, Lee et al. [3] identified the first miRNA gene, lin-4, in *Caenorhabditis elegans*. Since then, numerous studies have shown that miRNAs play an important role in life processes, including cell metabolism, proliferation, apoptosis, and development [4–8]. Besides, miRNAs are also involved in the occurrence and development of many *Human* diseases, such as prostatic neoplasms, breast neoplasms, and so on [9–11]. Therefore, identifying potential miRNA-disease associations is crucial in the research and treatment of *Human* diseases. Traditional experimental methods have high accuracy in predicting the miRNA-disease associations, but such methods are often limited to the disadvantages of small scale, high time-consuming, and high cost. Hence, using computational methods to predict the potential associations has gradually attracted more and more researchers [12, 13].

In the past few years, there are many computational methods have been developed to predict the miRNA-disease associations. For example, Chen et al. [14] developed a model named RBMMMDA, which utilizing the restricted Boltzmann machine to predict multi-type associations between miRNAs and diseases. This method can not only discover new potential associations between miRNAs and diseases but also indicate the corresponding association types. Chen et al. [15] proposed a novel method based on heterogeneous graph inference (HGIMDA). This approach takes advantage of the miRNA functional similarity, disease semantic similarity, Gaussian interaction profile kernel similarity, and known miRNA-disease associations. It breaks through the limitations of traditional methods and can be used for new miRNAs and diseases without any known associations. You et al. [16] constructed a heterogeneous graph and utilized the depth-first search algorithm (PBMDA). Compared with other previous models, this method has better reliability and accuracy. Chen et al. [17] proposed a new method of within and between score, named WBSMDA. This method can be used for diseases without any known related miRNAs. Wang et al. [18] proposed a method of the logistic model tree (LMTRDA) by combining miRNA sequence information, miRNA functional similarity, and disease semantic similarity. Li et al. [19] designed a novel method (MCMDA) for the prediction of potential miRNA-disease associations by updating the known association adjacency matrix. Zheng et al. [20] developed a prediction model based on the machine learning method. This model combines Gaussian interaction spectrum kernel similarity information, disease semantic similarity, and miRNA functional similarity and sequence information. Furthermore, it respectively utilizes the auto-encoder neural network (AE) and random forest for feature extraction and training. Zheng et al. [21] developed a novel model based on the distance sequence similarity method (DBMDA). This method utilizes the regional distance to calculate the global similarity and is implemented through a chaotic game representation algorithm based on miRNA sequences, which provides a new idea for the field of miRNA-disease prediction. Zeng et al. [22] summarized the calculation methods for predicting the potential associations between miRNA and disease based on biological interaction networks. By discussing the advantages and disadvantages of these methods, they provided constructive help for this problem. Zou et al. [23] developed two miRNA-disease association computational methods, one method uses social network analysis methods and machine learning, and the other is supervised machine learning methods, both of which have achieved excellent prediction results. Zeng et al. [24] constructed a heterogeneous network by integrating the neighborhood information in the neural network to predict the miRNA-disease associations (NNMDA). By comparing with other methods, the prediction performance of NNMDA is more accurate and reliable.

**Fig. 1:**
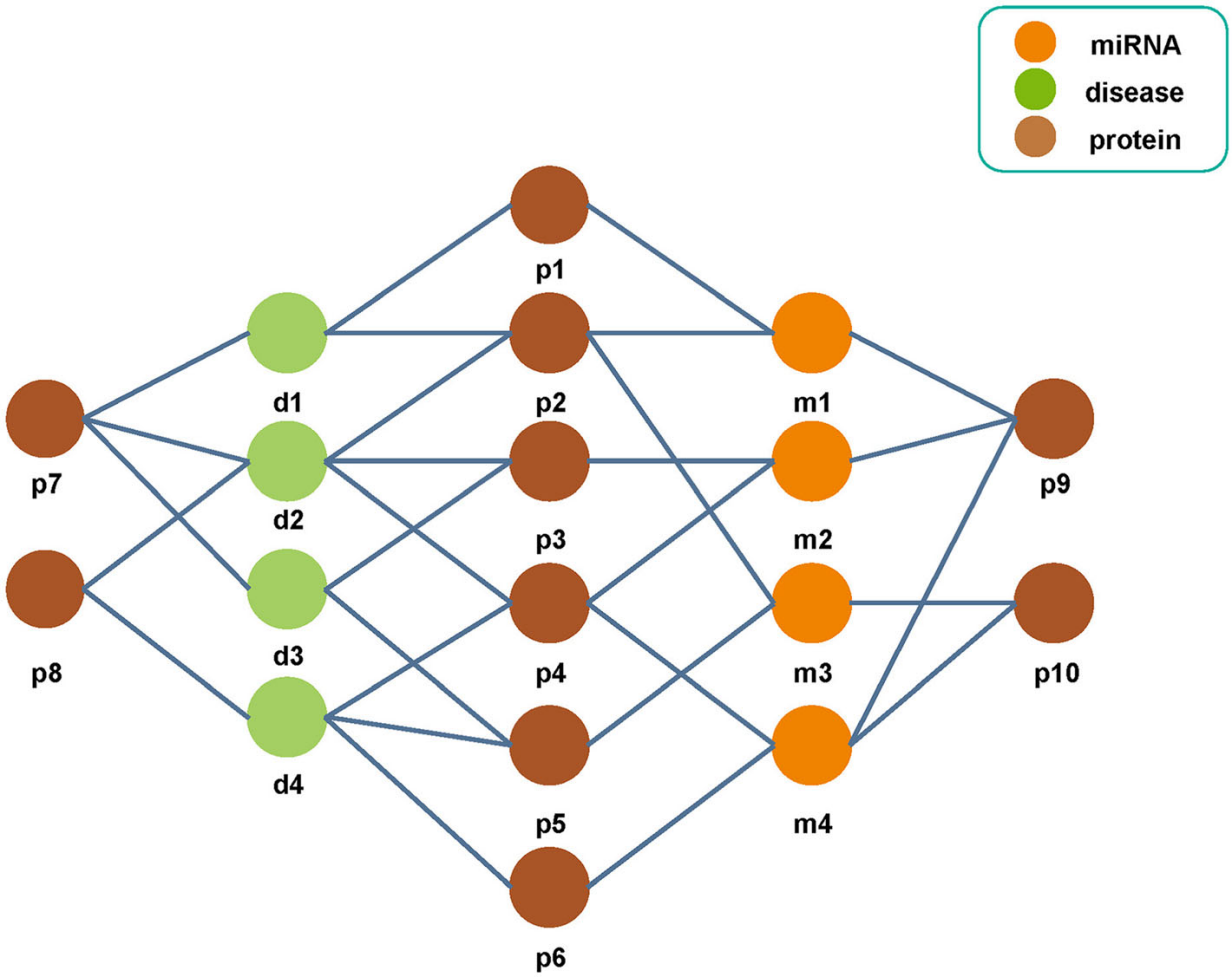
The miRNA-protein-disease network. The association network is constructed by combining the known miRNA-protein and protein-disease associations. Each node respectively represents miRNA, protein and disease, and each edge represents the relationship between the two biomolecules

**Fig. 2:**
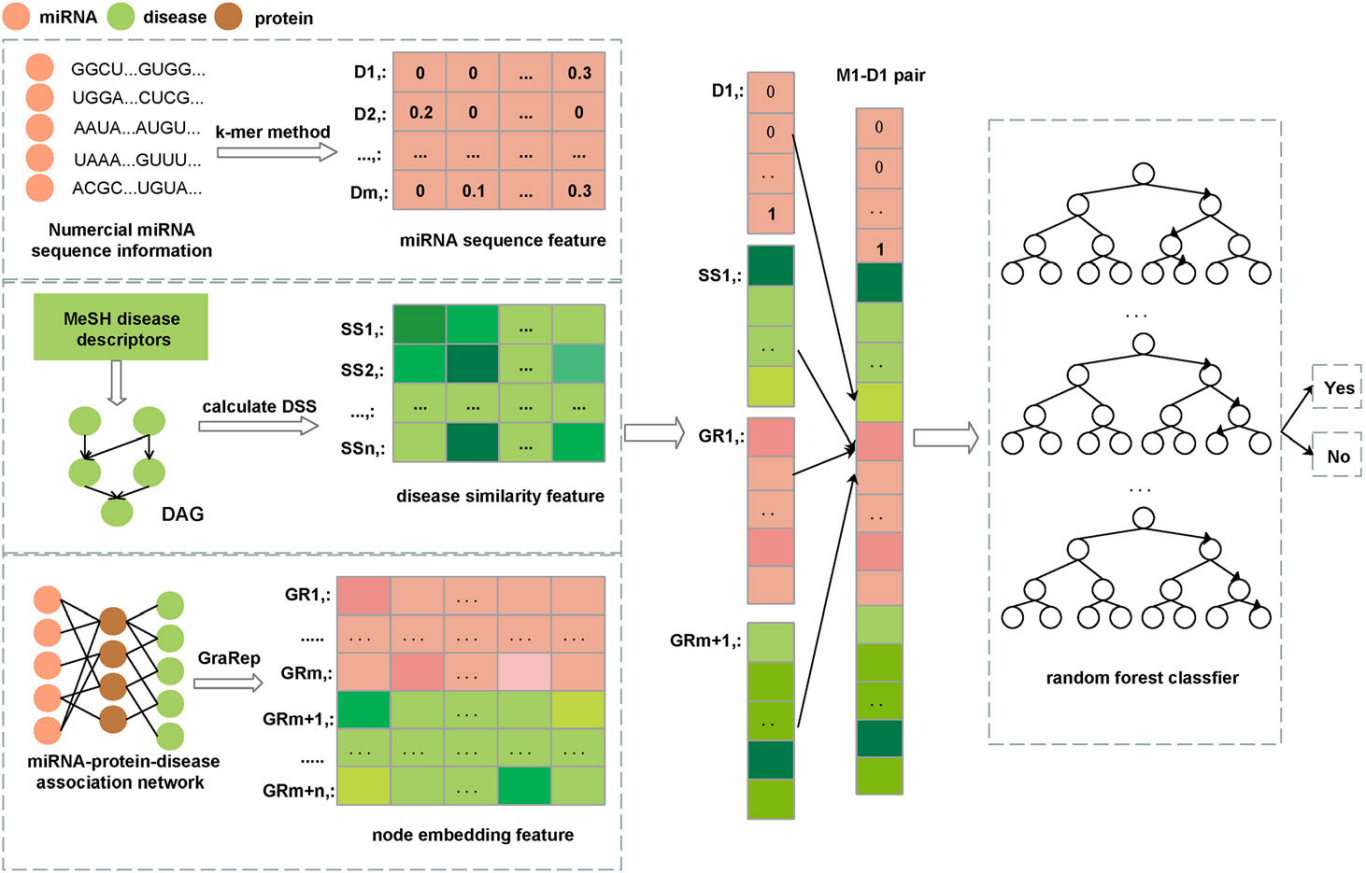
The pipeline of NEMPD. DSS represents disease semantic similarity

At present, most existing state-of-the-art algorithms only make use of the single known miRNA-disease associations for potential miRNA-disease association prediction. However, diseases are mainly caused by the disturbance of a complex of interacting multiple biomolecules, rather than the abnormity of a single biomolecule. In addition, the functionally dependent molecular components in *Human* cells form a complex biological network, in which proteins are an important part of *Human* tissues and cells. The protein-miRNA associations and protein-disease associations have been confirmed by many previous experiments [25–27]. Therefore, we proposed a novel method to predict the miRNA-disease associations by preserving behavior and attribute information based on the heterogeneous miRNA-protein-disease network and the GraRep network embedding method (NEMPD). More specifically, we firstly constructed and comprehensively analyzed a heterogeneous miRNA-protein-disease network by integrating the miRNA-protein and protein-disease associations (see Fig. 1). Secondly, the network representation method can be used to get the embedding representation of nodes from the network while maintaining the network property. In recent years, network embedding methods such as LINE [28], DeepWalk [29] and so on, have been applied to several bioinformatics problems and have good performance. In this article, we choose the GraRep [30] method to learn the association information with proteins (behavior information) of miRNAs and diseases. Thirdly, the behavior information of miRNAs and diseases is combined with their own attribute information (disease semantic similarity and miRNA sequence information) to represent the 16,427 known miRNA-disease pairs downloaded from HMDD [31] database. Finally, the Random Forest classifier was utilized to train the converted miRNA-disease feature pairs. The pipeline of NEMPD is shown in Fig. 2. In the experimental results, under five-fold cross-validation, the average AUC and AUPR of NEMPD is respectively 0.9158 and 0.9233. Furthermore, we measured the performance of NEMPD with different feature combinations and classifiers. Besides, in order to further test the performance of NEMPD, we conducted case studies of three major *Human* diseases. All the results demonstrate that NEMPD has a good performance and can be used as a reliable model in the field of miRNA-disease association prediction.

## Results and discussion

### The five-fold cross-validation performance of NEMPD

To evaluate the prediction performance of NEMPD, we adopted the 5-fold cross-validation method in our experiment. Specifically, we firstly divide the training set into five parts, where the ratio of positive and negative samples is the same in each part. Each time we select 4 parts as the training sample and the remaining 1 part as the test sample, and then repeat the experiment 5 times. In the results, we selected six parameters as evaluation indicators: accuracy (Acc.), precision (Prec.), matthews correlation coefficient (MCC), specificity (Spec.), sensitivity (Sen.) and areas under the ROC curve (AUC). Table 1 shows the results of each fold in detail. The final results well proved the good performance of NEMPD in the prediction of potential miRNA-disease associations.

**Table 1:** The 5-fold cross-validation performance of NEMPD

Fold	ACC.(%)	Spec.(%)	Sen.(%)	MCC(%)	Prec.(%)	AUC(%)
0	85.33	89.17	81.50	70.87	88.27	91.72
1	85.01	89.90	80.13	70.36	88.80	90.70
2	85.47	90.23	80.71	71.26	89.20	91.50
3	85.73	90.17	81.28	71.74	89.21	92.06
4	85.50	89.83	81.18	71.27	88.86	91.93
**Average**	**85.41 ± 0.26**	**89.86 ± 0.42**	**80.96 ± 0.55**	**71.10 ± 0.52**	**88.87 ± 0.38**	**91.58 ± 0.54**

**Fig. 3:**
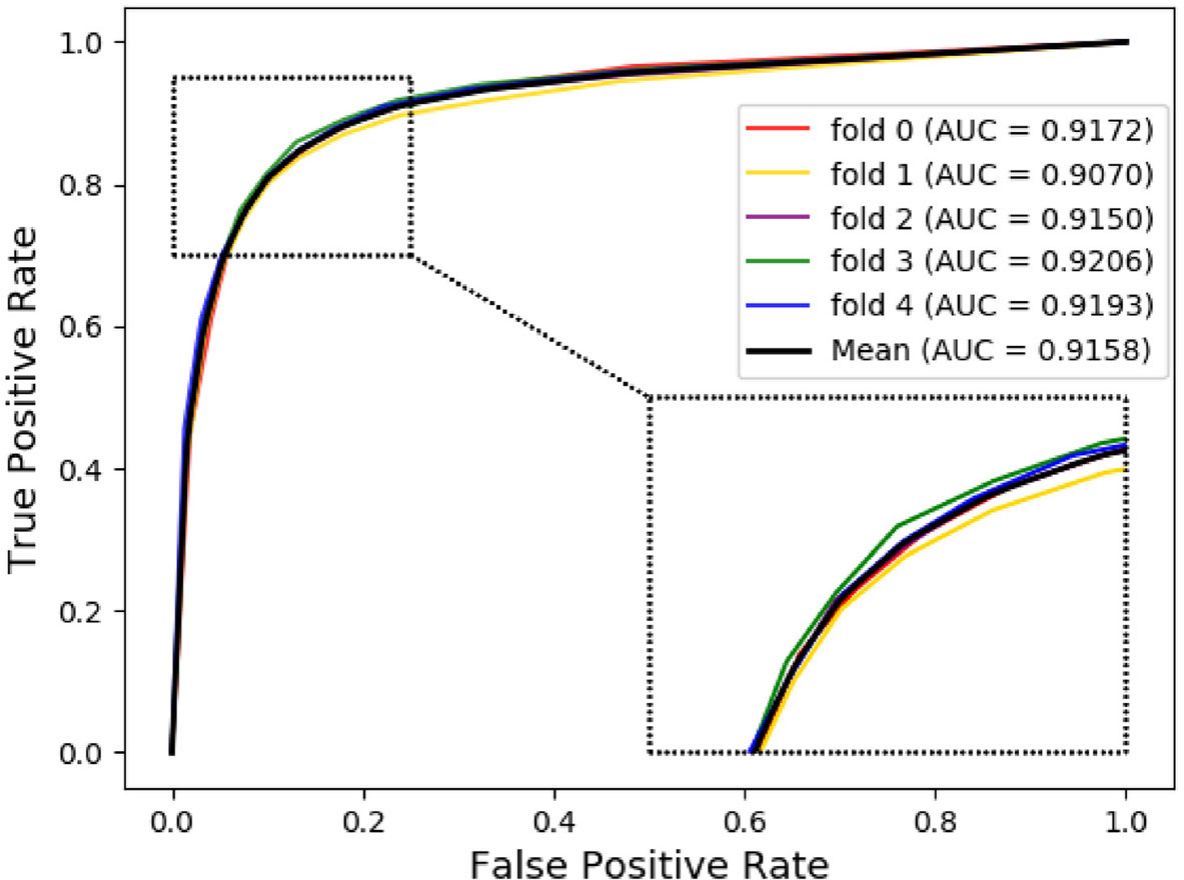
The 5-fold cross validation ROC curves and AUC of NEMPD

**Fig. 4:**
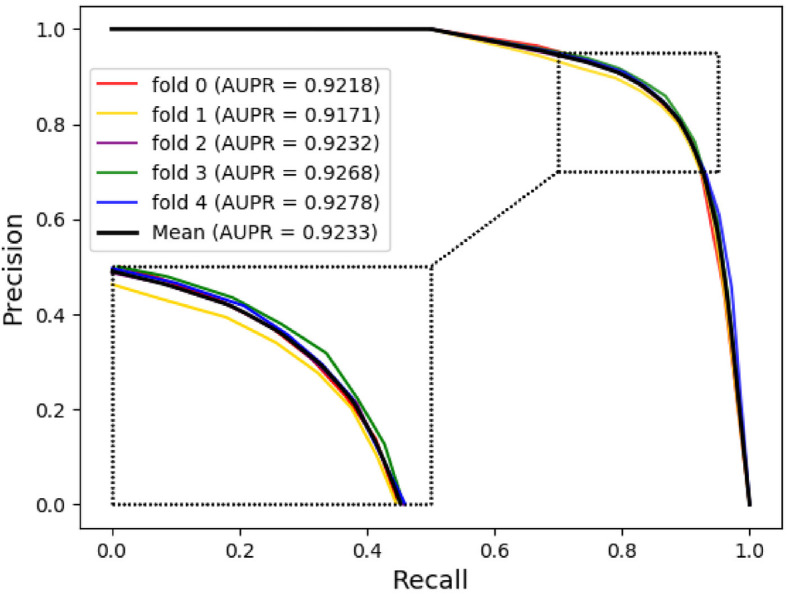
The 5-fold cross validation PR curves and AUPR of NEMPD

The ROC (Receiver Operating Characteristic) curve is often used to evaluate the advantages and disadvantages of a binary classifier, and to measure the non-equilibrium in classification. The abscissa of the ROC curve is FPR (false positive rate), which means the number of cases predicted to be positive among all negative cases. The ordinate of the ROC curve is TPR (true positive rate), which means the total predicted true positive samples. The AUC is defined as the areas under the ROC curve, with values generally ranging from 0.5 to 1. In general, the reason why AUC is usually used as an evaluation indicator in most cases is that the ROC curve cannot clearly indicate which classifier has a better effect. The PR (Precision-Recall) curve is another tool for evaluating the classification ability of machine learning algorithms for a given data set. Moreover, when dealing with some highly imbalanced data sets, the PR curve can display more information and find more problems. Similarly, the AUPR is defined as the areas under the PR curve. The ROC and PR curves of NEMPD under 5-fold cross-validation are respectively shown in Figs. 3 and 4. As we can be seen from the figure, the mean AUC and AUPR of NEMPD is 0.9158 and 0.9233, respectively. Generally, the results fully demonstrate that NEMPD has a good performance in the field of potential miRNA-disease association prediction.

### Comparison with different feature combinations

**Fig. 5:**
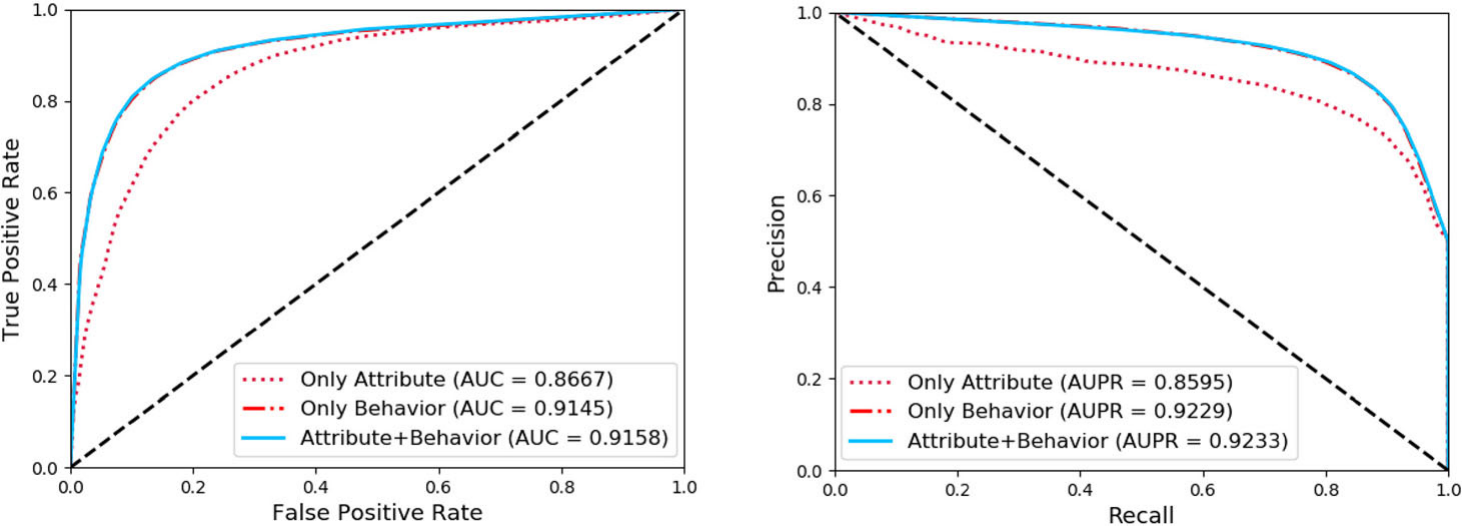
The ROC and PR curves of NEMPD with different combinations. Three different feature combinations (only attribute information, only behavior information, attribute and behavior information) were respectively used as training features of the random forest classifier and verified under 5-fold cross-validation

In order to verify the validity of the proposed feature representation information, we discussed the influence of different feature combinations on the results of NEMPD. In detail, the combination 1 is only composed of the attribute information of miRNAs and diseases, the combination 2 is only composed of behavior information of miRNAs and diseases, the combination 3 is composed of attribute and behavior information. These three different feature combinations were respectively used as training features of the random forest classifier and were verified under 5-fold cross-validation. The detailed results and ROC and PR curves are respectively shown in Table 2 and Fig. 5. In the end, the experimental results show that using the combination of attribute and behavior information as the final training feature vector can get better performance in the prediction.

**Table 2:** Performance of NEMPD with different combinations. Combination1 represents only attribute information. Combination2 represents only behavior information. Combination3 represents a combination of attribute and behavior information

	Acc.(%)	Spec.(%)	Sen.(%)	MCC(%)	Prec.(%)	AUC(%)
combination1	79.95 ± 0.68	78.25 ± 0.66	**81.65 ± 1.22**	59.95 ± 1.37	78.97 ± 0.55	86.67 ± 0.61
combination2	85.26 ± 0.52	89.57 ± 0.59	80.96 ± 0.73	70.79 ± 1.02	88.59 ± 0.60	91.45 ± 0.50
combination3	**85.41 ± 0.26**	**89.86 ± 0.42**	80.96 ± 0.55	**71.10 ± 0.52**	**88.87 ± 0.38**	**91.58 ± 0.54**

*combination1: only attribute information

*combination2: only behavior information

*combination3: attribute and behavior information

### Comparison with different classifier models

**Fig. 6:**
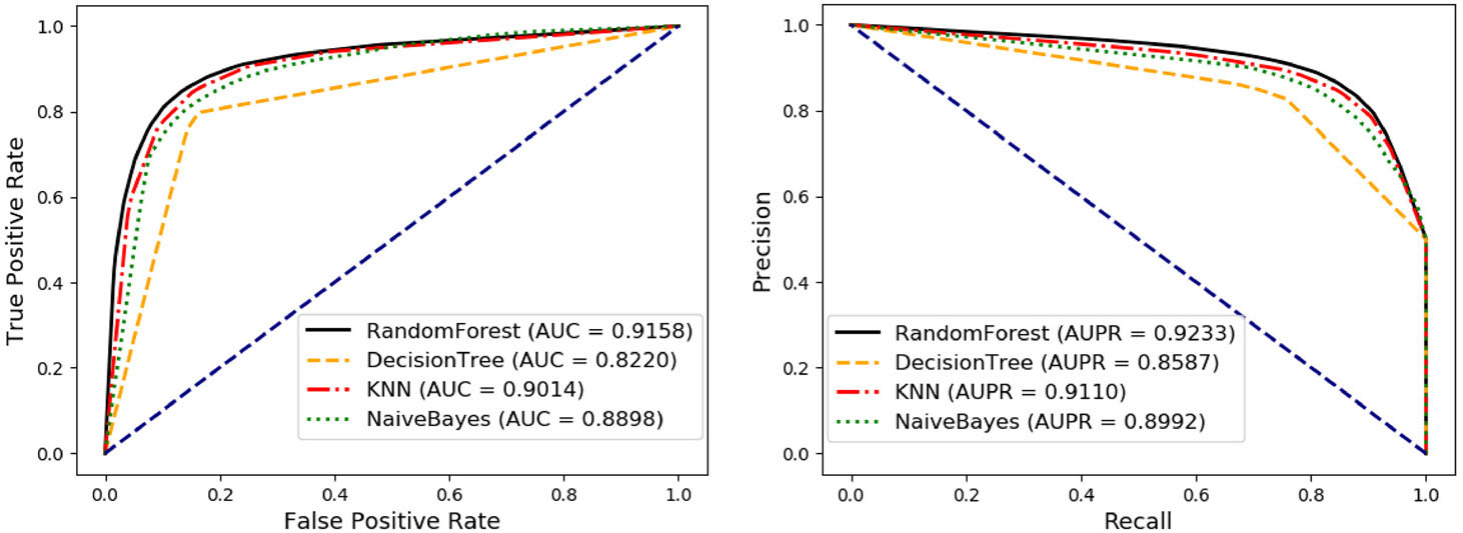
The ROC and PR curves of NEMPD with different classifiers

To verify the performance of the random forest classifier in NEMPD, we further compared it with three other different classifier models (KNN, Naive Bayes and Decision Tree). It is worth noting that all these four classifiers use the same data set, and all use the default parameters for training and prediction to ensure the effectiveness of the comparison. We also utilize these six parameters (accuracy (Acc.), precision (Prec.), matthews correlation coefficient (MCC), specificity (Spec.), sensitivity (Sen.) and areas under the ROC curve (AUC)) as evaluation indicators. As a result, the KNN model achieves the average AUC of 90.14 ± 0.48%, which the AUC value of each fold is 89.86, 89.52, 90.12, 90.73, and 90.47%. The Naive Bayes model achieves the average AUC of 88.98 ± 0.44%, which the AUC value of each fold is 88.79, 88.52, 88.84, 89.69, and 89.07%. The Decision Tree model achieves the average AUC of 82.20 ± 0.80%, which the AUC value of each fold is 81.66, 81.07, 82.59, 82.96, and 82.70%. The Random Forest model achieves the average AUC of 91.58 ± 0.54%, which the AUC value of each fold is 91.72, 90.70, 91.50, 92.06, and 91.93%. Details of the remaining 5 parameters are shown in Table 3, and Fig. 6 shows the ROC and PR curves of different classifiers. The results of the comparison experiment fully prove that the random forest classifier is more suitable for NEMPD. Although it is not as good as KNN and Naive Bayes in sensitivity, random forest performs better in accuracy and AUC, which can better reflect the classification ability of a model.

**Table 3:** Comparison of NEMPD with different classifiers

Classifier	ACC.(%)	Spec.(%)	Sen.(%)	MCC.(%)	Prec.(%)	AUC.(%)
KNN	84.71 ± 0.53	84.39 ± 0.71	**85.03 ± 0.59**	69.42 ± 1.07	84.49 ± 0.63	90.14 ± 0.48
Naive Bayes	83.04 ± 0.53	82.73 ± 0.66	83.34 ± 0.95	66.08 ± 1.05	82.84 ± 0.53	88.98 ± 0.44
DecisionTree	82.20 ± 0.80	84.96 ± 1.21	79.43 ± 0.96	64.50 ± 1.61	84.09 ± 1.09	82.20 ± 0.80
**RandomForest**	**85.41 ± 0.26**	**89.86 ± 0.42**	80.96 ± 0.55	**71.10 ± 0.52**	**88.87 ± 0.38**	**91.58 ± 0.54**

### Case studies

To further verify NEMPD’s ability to discover potential miRNA-disease associations, we selected three common *Human* diseases (colon neoplasms, breast neoplasms, and lung neoplasms) to conduct the case studies, which is the most common experiment in miRNA-disease association prediction methods. After the experiment was completed, we selected the top 50 predicted associations between miRNAs and corresponding cancers and confirmed them with two other databases, dbDEMC [32] and miR2Disease [33].

Colon neoplasms are currently the third common gastrointestinal disease in the world [34, 35]. Furthermore, some of the potential miRNA-colon neoplasms associations have been verified by previous experiments, such as miR-17, miR-92a, miR-31, miR-155, and miR-21 [36]. These researches have demonstrated that miRNA is crucial for the prediction of colon neoplasms and can be used as an important biomarker for colon neoplasms. Therefore, the prediction of miRNA-colon neoplasms associations is very important for the treatment and diagnosis of colon neoplasms. In this work, we sorted the final prediction results of NEMPD according to the prediction score. Finally, 48 of the top 50 miRNAs are verified to be associated with colon neoplasms through the miR2Disease and dbDEMC databases (see Table 4). For example, hsa-miR-20a-5p has been experimentally confirmed to be associated with colon neoplasms [37]. This method draws conclusions through statistical analysis of population-based colorectal cancer studies conducted in Utah and the Kaiser Permanente Medical Care Project (PMID: 26963002).

**Table 4:** The top 50 miRNAs associated with colon neoplasms were predicted by NEMPD. The top 1–25 associated miRNAs were shown in the first column. The top 26–50 associated miRNAs were shown in the third column

miRNA	Evidence	miRNA	Evidence
hsa-mir-20a-5p	dbDemc	hsa-mir-128-3p	Unconfirmed
hsa-mir-146a-5p	dbDemc	hsa-mir-125b-5p	dbDemc
hsa-mir-93-5p	dbDemc	hsa-mir-122-5p	dbDemc
hsa-mir-150-5p	dbDemc	hsa-mir-107	dbDemc;miR2Disease
hsa-mir-1-3p	dbDemc	hsa-mir-106b-5p	dbDemc
hsa-mir-429	dbDemc	hsa-mir-106a-5p	dbDemc
hsa-mir-133b	dbDemc;miR2Disease	hsa-mir-98-5p	dbDemc
hsa-mir-34a-5p	dbDemc	hsa-let-7 g-5p	dbDemc
hsa-mir-326	dbDemc	hsa-let-7c-5p	dbDemc
hsa-mir-96-5p	dbDemc	hsa-let-7a-5p	dbDemc
hsa-mir-29b-3p	dbDemc	hsa-mir-17-5p	dbDemc;miR2Disease
hsa-mir-26a-5p	dbDemc	hsa-mir-138-5p	dbDemc
hsa-mir-24-3p	dbDemc	hsa-mir-20b-5p	dbDemc
hsa-mir-21-5p	dbDemc	hsa-mir-216a-5p	dbDemc
hsa-mir-206	dbDemc;miR2Disease	hsa-mir-182-5p	dbDemc
hsa-mir-204-5p	dbDemc	hsa-mir-28-5p	dbDemc
hsa-mir-195-5p	dbDemc	hsa-mir-125a-5p	dbDemc
hsa-mir-181c-5p	dbDemc	hsa-mir-224-5p	dbDemc
hsa-mir-181b-5p	dbDemc	hsa-mir-424-5p	dbDemc
hsa-mir-181a-5p	dbDemc	hsa-mir-7-5p	Unconfirmed
hsa-mir-16-5p	dbDemc	hsa-mir-140-5p	dbDemc
hsa-mir-15b-5p	dbDemc	hsa-mir-18b-5p	dbDemc
hsa-mir-15a-5p	dbDemc	hsa-mir-18a-5p	dbDemc
hsa-mir-155-5p	dbDemc	hsa-mir-135a-5p	dbDemc
hsa-mir-145-5p	dbDemc	hsa-mir-34c-5p	dbDemc

Breast neoplasms are another common malignant tumor that mainly occurs in women. In the United States, there are about 180,000 new breast patients each year, and about 40,000 die from breast neoplasms. In recent years, the incidence of breast neoplasms in China is also rising and has become the second leading cause of cancer death after lung neoplasms. As a small molecule RNA, miRNA can inhibit breast neoplasms by inhibiting its target mRNA. Besides, the miRNA-breast neoplasms associations have been verified by many previous works of literature. For example, miR-21 has been found to be excessive in breast neoplasms [38], while miR-429 and miR-200c are down-regulated [39]. Similarly, we sorted the final prediction results according to the prediction score. Finally, 47 of the top 50 miRNAs are verified to be associated with breast neoplasms through the miR2Disease and dbDEMC databases (see Table 5). For example, hsa-miR-93-5p has been experimentally proved to be related to breast neoplasms [40] (PMID: 24865188).

**Table 5:** The top 50 miRNAs associated with breast neoplasms were predicted by NEMPD. The top 1–25 associated miRNAs were shown in the first column. The top 26–50 associated miRNAs were shown in the third column

miRNA	Evidence	miRNA	Evidence
hsa-mir-20a-5p	dbDemc	hsa-mir-155-5p	dbDemc
hsa-mir-503-5p	dbDemc	hsa-mir-18a-5p	dbDemc
hsa-mir-93-5p	dbDemc	hsa-mir-145-5p	dbDemc
hsa-mir-9-5p	dbDemc	hsa-mir-128-3p	dbDemc
hsa-mir-661	dbDemc;miR2Disease	hsa-mir-125b-5p	dbDemc
hsa-mir-532-5p	dbDemc	hsa-mir-122-5p	Unconfirmed
hsa-mir-429	dbDemc;miR2Disease	hsa-mir-107	dbDemc;miR2Disease
hsa-mir-34b-5p	dbDemc	hsa-mir-106b-5p	dbDemc
hsa-mir-424-5p	dbDemc	hsa-mir-106a-5p	dbDemc
hsa-mir-326	dbDemc	hsa-mir-100-5p	dbDemc
hsa-mir-7-5p	dbDemc	hsa-let-7 g-5p	dbDemc
hsa-mir-29b-3p	dbDemc	hsa-let-7c-5p	dbDemc
hsa-mir-26a-5p	dbDemc	hsa-let-7a-5p	Unconfirmed
hsa-mir-140-5p	dbDemc	hsa-mir-184	dbDemc;miR2Disease
hsa-mir-21-5p	dbDemc	hsa-mir-17-5p	dbDemc;miR2Disease
hsa-mir-206	dbDemc;miR2Disease	hsa-mir-138-5p	Unconfirmed
hsa-mir-204-5p	dbDemc	hsa-mir-20b-5p	dbDemc
hsa-mir-199b-5p	dbDemc	hsa-mir-324-5p	dbDemc
hsa-mir-195-5p	dbDemc	hsa-mir-135a-5p	dbDemc
hsa-mir-181c-5p	dbDemc	hsa-mir-34c-5p	dbDemc
hsa-mir-181b-5p	dbDemc	hsa-mir-182-5p	dbDemc
hsa-mir-181a-5p	dbDemc	hsa-mir-520 h	dbDemc
hsa-mir-16-5p	dbDemc	hsa-mir-28-5p	dbDemc
hsa-mir-15b-5p	dbDemc	hsa-mir-125a-5p	dbDemc
hsa-mir-15a-5p	dbDemc	hsa-mir-224-5p	dbDemc

Lung neoplasms are a common tumor disease worldwide and one of the leading causes of cancer death. It is also one of the fastest-growing morbidity and mortality rates and the most threatening to the health and life of the population. In recent years, the incidence and mortality of lung cancer in many countries have increased significantly. In addition, miRNAs have been confirmed by many previous researches that are crucial in the early treatment and diagnosis of lung neoplasms. For example, Yanaihara et al. [41] found that the expression of 17 miRNAs in lung cancer cells has changed compared to normal cells through microarray analysis. Mascaux et al. [42] also found that the expression profile of miRNAs also changed during the entire process of lung cancer. Similarly, we sorted the final prediction results of NEMPD according to the prediction score. Finally, 47 of the top 50 miRNAs were verified to be related to lung neoplasms by the dbDEMC and miR2Disease databases (see Table 6).

**Table 6:** The top 50 miRNAs associated with lung neoplasms were predicted by NEMPD. The top 1–25 associated miRNAs were shown in the first column. The top 26–50 associated miRNAs were shown in the third column

miRNA	Evidence	miRNA	Evidence
hsa-mir-20a-5p	dbDemc	hsa-mir-145-5p	dbDemc
hsa-mir-146a-5p	Unconfirmed	hsa-mir-128-3p	dbDemc
hsa-mir-93-5p	dbDemc	hsa-mir-125b-5p	dbDemc
hsa-mir-9-5p	dbDemc	hsa-mir-122-5p	Unconfirmed
hsa-mir-429	dbDemc;miR2Disease	hsa-mir-107	dbDemc
hsa-mir-34b-5p	dbDemc	hsa-mir-106b-5p	dbDemc
hsa-mir-34a-5p	dbDemc	hsa-mir-106a-5p	dbDemc
hsa-mir-326	dbDemc	hsa-mir-100-5p	dbDemc
hsa-mir-31-5p	dbDemc	hsa-let-7 g-5p	dbDemc
hsa-mir-29b-3p	dbDemc	hsa-let-7c-5p	dbDemc
hsa-mir-26a-5p	dbDemc	hsa-let-7a-5p	dbDemc
hsa-mir-24-3p	dbDemc	hsa-mir-184	dbDemc
hsa-mir-21-5p	dbDemc	hsa-mir-17-5p	dbDemc;miR2Disease
hsa-mir-206	dbDemc	hsa-mir-138-5p	dbDemc
hsa-mir-204-5p	dbDemc	hsa-mir-140-5p	dbDemc
hsa-mir-199b-5p	dbDemc	hsa-mir-324-5p	dbDemc;miR2Disease
hsa-mir-195-5p	dbDemc	hsa-mir-942-5p	dbDemc
hsa-mir-181c-5p	dbDemc	hsa-mir-182-5p	dbDemc
hsa-mir-181b-5p	dbDemc	hsa-mir-520 h	dbDemc
hsa-mir-181a-5p	dbDemc	hsa-mir-28-5p	dbDemc
hsa-mir-16-5p	dbDemc	hsa-mir-125a-5p	dbDemc;miR2Disease
hsa-mir-15b-5p	dbDemc	hsa-mir-224-5p	dbDemc
hsa-mir-15a-5p	dbDemc	hsa-mir-503-5p	dbDemc
hsa-mir-155-5p	Unconfirmed	hsa-mir-424-5p	dbDemc
hsa-mir-153-3p	dbDemc	hsa-mir-7-5p	dbDemc

## Conclusion

The prediction of potential miRNA-disease associations by using computational models addresses the disadvantages of high time-consuming and cost of traditional methods, and provides data support for traditional experimental researches. In this article, we proposed a novel computational model (NEMPD) by constructing a heterogeneous miRNA-protein-disease network based on known miRNA-protein and protein-disease associations and utilizing the GraRep network embedding method to obtain network behavior information (association information with proteins) of miRNA and disease. After that, their intrinsic attribute and behavior information are combined into the final node feature vectors. Finally, the converted known miRNA-disease pairs are used for training and prediction by the random forest classifier. In the results, NEMPD obtained the average AUC and AUPR values of 0.9158 and 0.9233 under 5-fold cross-validation. Moreover, we also verified colon neoplasms, breast neoplasms, and lung neoplasms for case studies, and respectively confirmed 48, 47, and 47 miRNAs in the top 50 prediction results. All the experimental results proved that NEMPD can effectively predict potential miRNA-disease associations and can also be extended to other biological small molecule association prediction researches.

## Methods

### Construct the miRNA-protein-disease association network

The miRNA-protein-disease association network is constructed by combining the known miRNA-protein and protein-disease associations. More specifically, the miRNA-protein and protein-disease associations are respectively obtained from the miRTarBase [43] and DisGeNET database [44]. After that, we unified identifiers and simplified unrelated items. Finally, a total of 4944 groups of miRNA-protein associations and 25,087 groups of protein-disease associations were acquired (see Table 7). In addition, we further classified the three types of nodes in the network and separately calculate the number of them. Finally, 271 miRNA nodes, 1147 protein nodes and 693 disease nodes were respectively got (see Table 8).

**Table 7:** The associations in the miRNA-protein-disease network

Association type	Database	Amount
miRNA-protein	miRTarBase	4944
protein-disease	DisGeNET	25,087
Total	N/A	30,031

**Table 8:** The nodes in the miRNA-protein-disease network

Node	Amount
MiRNA	271
Disease	693
Protein	1147
Total	2111

### Numerical miRNA sequence information

In this work, the numerical miRNA sequence information derived from the miRbase [45] database was used as its own attribute information. At the same time, considering the simplicity of the experiment, we choose the 3-mer method to encode the miRNA sequences into 64(4 × 4 × 4) dimension vectors, where each dimension means the occurrence rate of the corresponding 3-mer of miRNA sequences (e.g. UGA, AGC, CUA).

### Disease semantic similarity

**Fig. 7:**
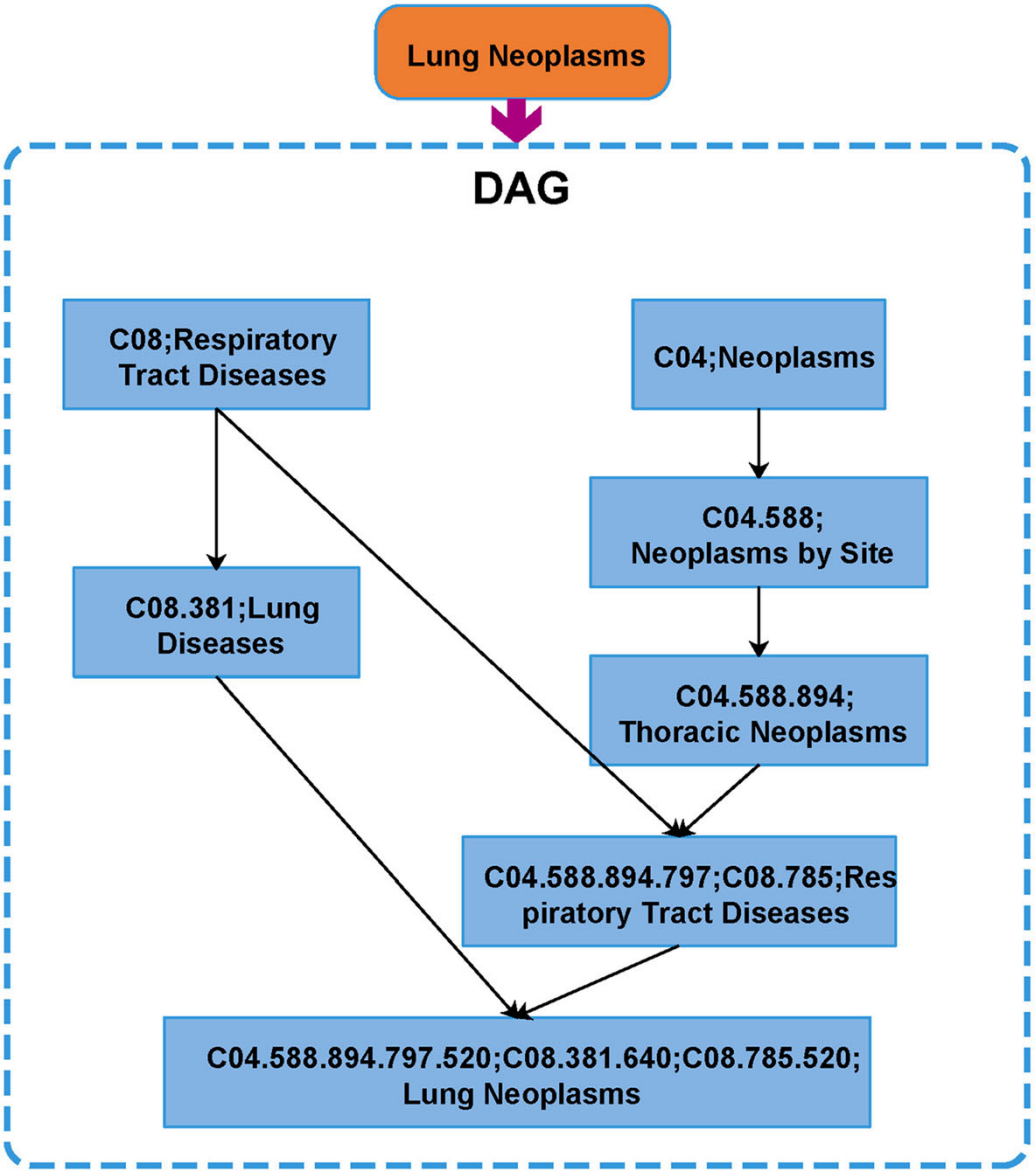
The DAGs of lung neoplasms. DAGs represents direct acyclic graphs

Disease semantic similarity has been widely used in the identification of disease-related miRNAs, and its effectiveness has been fully proved in a large number of previous studies [46–50]. Therefore, we choose to use disease semantic similarity to represent the attribute information of disease and calculate it based on its direct acyclic graphs (DAGs) and MeSH descriptors. For example, disease C can be described as DAG(C) = (D(C), E(C)), where D(C) is composed of the disease itself and its ancestor, and E(C) is composed of all edges from the parent node to the child node. Figure 7 below shows the DAG of lung neoplasms.

In traditional calculation models [46], disease terms at the same layer contribute the same semantic value to diseases. In fact, it is inaccurate to assign the same contribution value to two disease items on the same layer because they appear differently in the DAGs. In this article, we calculate the contribution of disease to the semantic value of disease C based on the assumption that the more specific diseases should contribute more to the semantic value of disease C. In this way, the contribution of a disease *d* to DAG(C) can be defined as follows:

1 $$ \left\{\begin{array}{c}{\mathrm{C}}_{\mathrm{C}}\left(\mathrm{d}\right)=1\ \mathrm{if}\ \mathrm{d}=\mathrm{C}\\ {}{\mathrm{C}}_{\mathrm{C}}\left(\mathrm{d}\right)=\max \left\{\Delta  \ast {\mathrm{C}}_{\mathrm{C}}\left({\mathrm{d}}^{\prime}\right)|{\mathrm{d}}^{\prime}\in \mathrm{children}\ \mathrm{of}\ \mathrm{d}\right\}\ \mathrm{if}\ \mathrm{d}\ne \mathrm{C}\end{array}\right. $$

where ∆ is the semantic contribution factor. Therefore, the semantic value of disease C can be obtained by adding the contributions of all ancestor diseases and disease *d* itself:

2 $$ \mathrm{C}\left(\mathrm{C}\right)={\sum}_{d\in DAG(C)}{C}_C(t) $$

Besides, the semantic similarity between disease A and disease B can be obtained by adding together the contributions of disease terms shared by the two disease DAGs:

3 $$ \mathrm{SS}\left(\mathrm{A},\mathrm{B}\right)=\frac{\sum_{d\in D(A)\cap D(B)}\left({C}_A(d)+{C}_B(d)\right)}{C(A)+C(B)} $$

### GraRep network embedding model

In many practical problems, information is usually organized using graphs, so it is important to learn useful information from graphs. One strategy for learning graph representations is that each node of the graph is represented by a low-dimensional vector, which contains meaningful semantic, relational, and structural information. GraRep [30] is one of these network embedding models for learning vector representations of weighted graph nodes. It utilizes low-dimensional vectors to represent the node vectors which appear in the graph, and integrate the global structure information of the graph into the learning process. By operating different global transformation matrices defined in the graph, GraRep can directly obtain the *k*-order relation information between nodes without involving a slow and complicated sampling process. Besides, different loss functions are used to capture different *k*-order local relation information, and matrix decomposition technology is used to optimize each model. In this way, the global representation of each vertex is constructed by combining different representations obtained from different models. This learned global representation can be used as a feature for further processing. More specifically, the basic steps of the whole algorithm are as follows:

- Step 1. Get *k*-step transition probability matrix *A*^*k*^, where *k* = 1,2...K.

Given the graph G, we can calculate the *k*-step transition probability matrix *A*^*k*^ by the product of the inverse of the degree matrix D and the adjacent matrix S (for weighted graphs, S is a real matrix; for unweighted graphs, S is a binary matrix).

- Step 2. Get each *k*-step representation.

Get the *k*-step log probability matrix *X*^*k*^, and minus the log(β) of each term, and replace the negative terms with 0. Then, construct the row representation vector of *W*^*k*^. Finally, the *k*-step representation of each node in the graph was obtained.

- Step 3. Connect all *k*-step representations.

All the *k*-step representations are linked together to form a global representation, which can be used as features in other tasks.

Table 9 describes the whole algorithm in detail.

**Table 9:** The GraRep overall algorithm

**GraRep Algorithm**	
**Input**	
Adjacency matrix S on graph	
Maximum transition step K	
Log shifted factor β	
Dimension of representation vector d	
**1. Get k-step transition probability matrix** ***A***^***k***^	
Compute A = *D*^−1^*S*	
Calculate *A*^−1^, *A*^−2^, *A*^−3^, …, *A*^*k*^, respectively	
**2. Get each k-step representations**	
For k = 1 to K	
2.1 Get positive log probability matrix	
calculate ${\Gamma}_1^k,{\Gamma}_2^k,{\Gamma}_3^k,\dots, {\Gamma}_N^k$ (${\Gamma}_i^k={\sum}_p{A}_{p,j}^k$) respectively	
calculate $\left\{{X}_{i,j}^k\right\}$	
${X}_{i,j}^k$ = log ($\frac{A_{i,j}^k}{\Gamma_j^k}$) – log(β)	
assign negative entries of *X*^*k*^ to 0	
2.2 Construct the representation vector *W*^*k*^	
SVD(*X*^*k*^) = [*U*^*k*^, ∑^*k*^, (V^k^)^*T*^]	
$$ {W}^k={U}_d^k\ {\left({\sum}_d^k\right)}^{\frac{1}{2}} $$	
End for	
**3. Concatenate all the k-step representations**	
W = [*W*^1^, *W*^2^, *W*^3^, …*W*^*k*^]	
**Output**	
Matrix of the graph representation W	

### Node representation

In order to improve the accuracy of the training results, we added the attribute information on the basis of the network behavior information of miRNAs and diseases to represent the final feature information of known miRNA-disease training pairs. Among them, the network behavior information of miRNA and disease nodes is extracted based on the miRNA-protein-disease network and the GraRep network embedding method. After that, we respectively select the sequence feature and semantic similarity information as the attribute feature of miRNA and disease. Finally, the known miRNA-disease training pairs are transformed into a 128-dimensional feature vector for training and prediction by using a random forest classifier.

## Data Availability

The datasets generated and/or analyzed during this study are available under open licenses in the data repository, https://github.com/jiboya123/NEMPD.

## Abbreviations

GraRep: Learning Graph Representations with Global Structural Information
AUC: the areas under the Receiver Operating Characteristic curve
AUPR: the areas under the Precision-Recall curve
DAGs: direct acyclic graphs
DSS: disease semantic similarity
HMDD: human microRNA disease database
